# Addressing flaws in the Seq2Topt dataset for the prediction of enzyme optimal temperature

**DOI:** 10.1093/bib/bbag282

**Published:** 2026-05-31

**Authors:** Peng Zhou

**Affiliations:** State Key Laboratory of Submarine Geoscience, Second Institute of Oceanography, Ministry of Natural Resources, No. 36 North Bao Chu Road, Xihu District, 310012 Hangzhou, China; Key Laboratory of Marine Ecosystem Dynamics, Ministry of Natural Resources and Second Institute of Oceanography, Ministry of Natural Resources, No. 36 North Bao Chu Road, Xihu District, 310012 Hangzhou, China

To the Editor,

I read the article from Qiu *et al.* with great interest, in which the authors developed a deep learning predictor, Seq2Topt, to estimate the optimal temperature (*T_opt_*) of enzymes. Seq2Topt achieved a prediction performance with an RMSE of 12.26°C using only protein sequence information [1]. Temperature is one of the critical factors governing enzyme catalytic activity [2]. A comprehensive understanding of *T_opt_* facilitates enzyme modification and industrial utilization through accurate *T_opt_* prediction. Accordingly, the development of Seq2Topt provides a promising computational tool for enzyme mining and *in silico* enzyme design.

Nevertheless, although Seq2Topt yielded an RMSE of 12.26°C, the predictive performance still has considerable room for improvement. Improved model accuracy would enhance its practical value and applicability in real industrial and research scenarios. As the overall performance of deep learning models is fundamentally determined by the quality of their underlying datasets, I conducted a careful data inspection and verification. The relevant raw files were downloaded from the corresponding GitHub repository of Seq2Topt (https://github.com/SizheQiu/Seq2Topt). The training set accounted for 90% of the total dataset. I sorted the data in the file named “train.csv” in ascending order of annotated *T_opt_* values and selected nine entries with low *T_opt_* values (<15°C) for literature cross-validation and manual inspection. Surprisingly, rigorous data verification revealed that all nine selected entries were problematic. These nine anomalous records are summarized in Table 1, and the underlying causes of the inconsistent or missing experimental evidence are discussed in detail below.

**Table 1:** Nine anomalous entries found from Seq2Topt Dataset.

UniProt accession number	*T_opt_* in Seq2Topt dataset (°C)	*T_opt_* from literature (°C)
F9FU71	0	Not found
A9U908	4	Not found
J7HET3	4	Not found
P94368	5	Not found
Q5YXQ1	7.5	Not found
Q83V33	10	Not found
Q50539	13	Not found
Q50538	13	Not found
Q2WCS9	13	37

F9FU71 is a linoleate diol synthase from *Fusarium oxysporum*. Its *T_opt_* was annotated as 0°C in the Seq2Topt training set [1]. Nevertheless, available experimental study only indicated incubation on ice during enzyme assay [3], with no credible evidence supporting a *T_opt_* of 0°C.

A9U908 and J7HET3 encode superoxide dismutase sodA and sodB in *Yersinia enterocolitica*, respectively. Both enzymes were assigned a *T_opt_* of 4°C in the Seq2Topt training set. Although high enzymatic activity at 4°C had been documented in the literature [4], no activity measurements at lower temperatures were available. Accordingly, the reliability of the annotated *T_opt_* of 4°C remains questionable.

P94368 is an ADP-dependent NAD(P)H-hydrate dehydratase from *Bacillus subtilis*, with a labeled *T_opt_* of 5°C in the Seq2Topt training set. After comprehensive literature retrieval [5–7], no experimental evidence or conclusive statement was found to confirm a *T_opt_* of 5°C for this enzyme.

Q5YXQ1 is a maleate isomerase from *Nocardia farcinica*, recorded with a *T_opt_* of 7.5°C in the Seq2Topt training set. However, no study has officially defined its *T_opt_* as 7.5°C; only an optimal pH of 7.5 for this enzyme was reported [8]. Hence, the authenticity of this *T_opt_* annotation requires further verification.

Q83V33 is an aminomuconate-semialdehyde dehydrogenase from *Pseudomonas fluorescens*. Seq2Topt annotated its *T_opt_* as 10°C. In the relevant study, enzyme activity assays were merely conducted at 10°C [9], whereas this temperature was not determined as the optimum. No experimentally validated *T_opt_* was found.

Q50538 and Q50539 represent two subunits of the corrinoid/iron–sulfur enzyme of the CO dehydrogenase complex from *Methanosarcina thermophila*, with a unified annotated *T_opt_* of 13°C in the Seq2Topt training set. Similar to Q83V33, the relevant literature only confirmed that multiple protein purification steps were carried out at 13°C for heterologously expressed proteins [10], rather than defining 13°C as their *T_opt_*.

Q2WCS9 is a glutamate dehydrogenase (GDH) from *Lactiplantibacillus plantarum*, annotated with a *T_opt_* of 13°C in the Seq2Topt training set. In contrast, a relevant study showed that the GDH activity of the cytoplasm fraction of *Lactiplantibacillus plantarum* strains tested reached its optimum at 37°C [11], revealing a remarkable data discrepancy.

In summary, all nine entries inspected were identified as anomalous through literature cross-checking. The Seq2Topt study stated that its dataset (*n* = 2917) was retrieved from the GitHub repository of TOMER [12], which was originally obtained from the BRENDA database [13]. Data quality fundamentally affects the training, optimization, and performance evaluation of machine learning models. To mitigate the above data-related issues, the existing *T_opt_* annotations should be thoroughly inspected and corrected. Upon careful verification of the *T_opt_* annotations, a more reliable dataset is expected.

I hope these remarks contribute to further refinement of enzyme *T_opt_* prediction models and promote their more reliable practical applications.

## Data Availability

No new data were generated in this study. The letter refers to the data provided by Seq2Topt study [1].
